# Femtosecond laser-assisted cataract surgery with implantation of a diffractive trifocal intraocular lens after laser in situ keratomileusis: a case report

**DOI:** 10.1186/s12886-018-0834-y

**Published:** 2018-07-03

**Authors:** Wei Wang, Shuang Ni, Xi Li, Xiang Chen, Yanan Zhu, Wen Xu

**Affiliations:** Eye Center of the 2nd Affiliated Hospital, School of Medicine, Zhejiang University, No.88 Jiefang Road, Hangzhou, China

**Keywords:** Femtosecond laser, Cataract surgery, Trifocal intraocular lens, Laser in situ keratomileusis

## Abstract

**Background:**

We report for the first time, a case of femtosecond laser-assisted cataract surgery (FLACS) with implantation of a diffractive trifocal intraocular lens (IOL) after laser in situ keratomileusis (LASIK).

**Case presentation:**

A 60-year-old man underwent FLACS uneventfully 15 years after myopic LASIK. An AT Lisa tri 839MP IOL was implanted with the expectation of spectacle independence. The Haigis-L formula was chosen for calculation of the IOL power and it provided reliable results. Three months postoperatively, the uncorrected visual acuities were 0.00 logMAR for distance, 0.10 logMAR for intermediate, and 0.10 logMAR for near.

**Conclusions:**

This case suggested that FLACS presents a feasible surgical technique for post-LASIK eyes and that implantation of trifocal IOL can achieve good visual performance in strictly selected cases after myopic LASIK.

## Background

There are a growing number of patients who wish to be spectacle independent after cataract surgery, and this includes some of the millions of people worldwide who have undergone laser in situ keratomileusis (LASIK). Ophthalmologists will encounter many cataract cases that have had previous LASIK surgery and they should have the knowledge to deal with these cases efficiently to achieve the best possible visual and refractive outcomes [1]. Femtosecond laser–assisted cataract surgery (FLACS) has become increasingly more common for its many advantages offered [2]. At the same time, various kinds of multifunctional intraocular lens (IOL) were designed [3] to provide functional visual restoration after cataract surgery. Nowadays, the combination of FLACS with multifocal IOLs has come to the cutting edge of cataract surgery. However, few studies had shed light on whether post-LASIK patients would benefit from this technique combo. Here we report a case of FLACS with the implantation of a diffractive trifocal IOL after LASIK.

## Case presentation

**Fig. 1:**
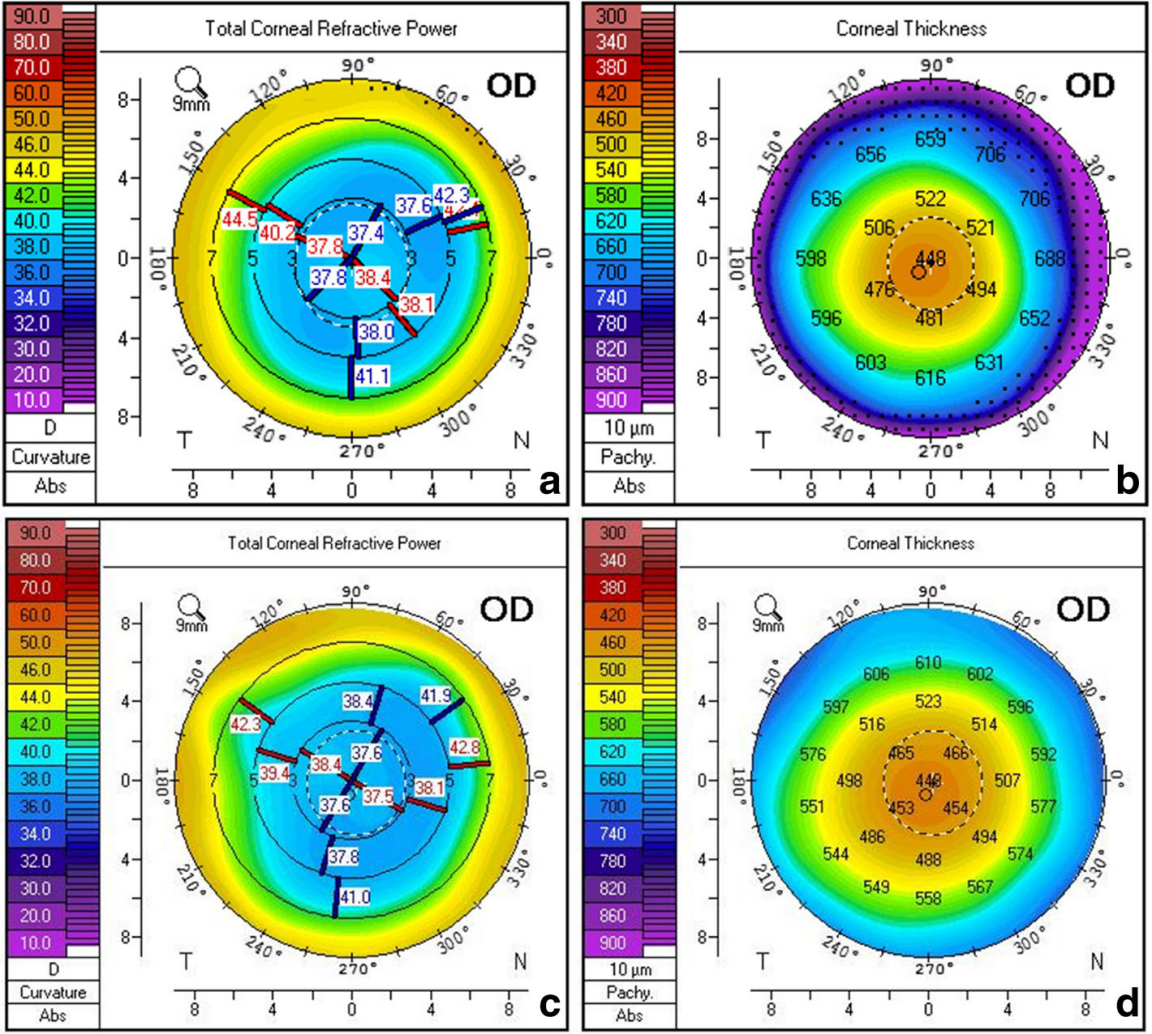
Corneal topography preoperatively (**a**, **b**) and at 3 months postoperatively (**c**, **d**)

A 60-year-old man was diagnosed with nuclear cataract in his right eye about 15 years after myopic LASIK surgery. His corrected distance visual acuity (CDVA) of the right eye was 0.52 logMAR with the refraction of − 4.50/− 0.75*29. He asked for a FLACS and desired spectacle independence after the IOL implantation. Corneal topography (Pentacam, Oculus Optikgerate GmbH, Wetzlar, Germany) showed a uniform, well-centered corneal flap (Fig. 1a, b), with a total corneal astigmatism of 0.9D, and a corneal irregular astigmatism of 0.115 μm. Besides, the 6 mm zone corneal spherical aberration (SA) was 0.392 μm while the angle kappa was 0.15. After a series of thorough assessments, we decided to implant a multifocal IOL with negative SA. For IOL power calculations, the standard IOLMaster (Carl Zeiss Meditec,Jena, Germany) biometry was performed and the Haigis-L formula was chosen to determine an IOL power of +23D for emmetropia. A steep merdian corneal incision was designed at 140 degree according to the Pentacam results.

**Fig. 2:**
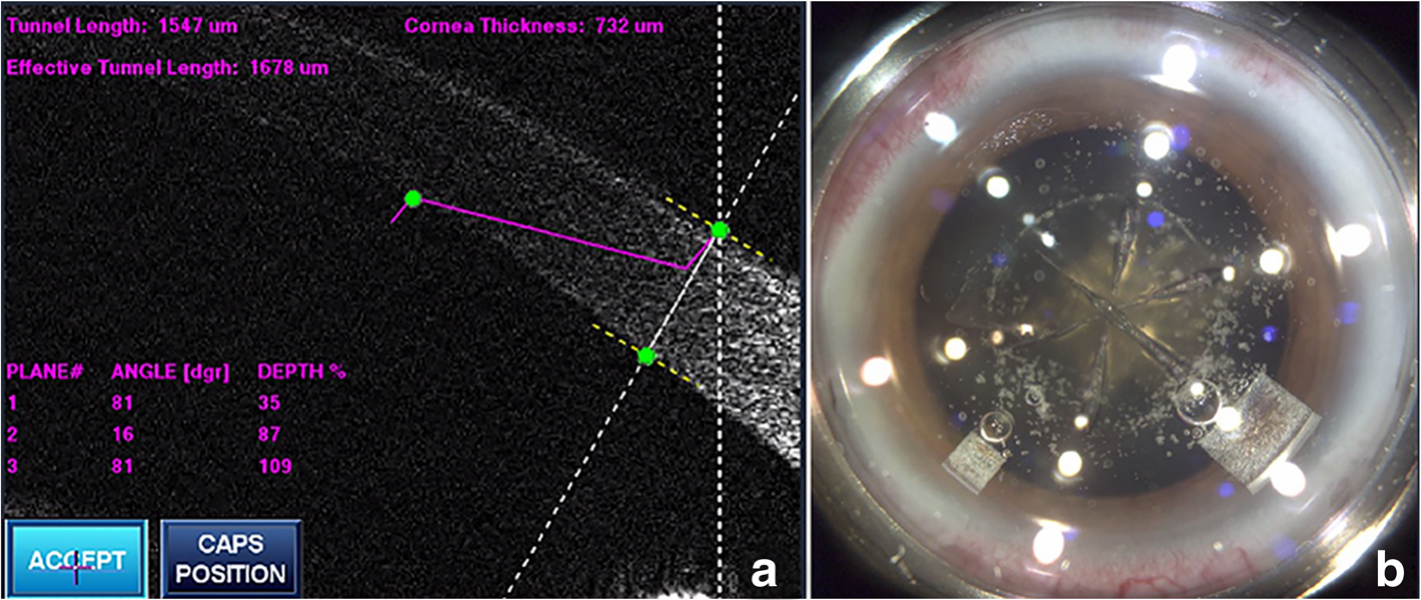
**a** AS-OCT image depicts the architecture of clear corneal incision. **b** Screenshot taken after femtosecond laser treatment completed. The primary corneal incision was made at 140 degree, consistent with the steep merdian axis

The LenSx laser system (LenSx Laser; Alcon Laboratories, Inc., Fort Worth,TX, USA) was used to perform the surgery. After the patient’s eye was properly docked to the system, the arc cuts of the primary and side port incision were adjusted towards the limbus, anterior to the conjunctival vascular arcades, under the guided of the LenSx real-time imaging system. A 2.0 mm primary corneal incision (Fig. 2a), a 1.0 mm side port incision and a 5.0 mm capsulotomy were created by the laser. Nuclear prefragmention was performed to obtain 6 pieces in a cross pattern (Fig. 2b). Then phacoemulsification was proceeded in a standard stop-and-chop manner with the Stellaris system (Bausch + Lomb Laboratories, Rochester, NY, USA), and an AT Lisa tri 839MP IOL (Carl Zeiss Meditec AG) was implanted right afterwards. All surgical procedures were uneventful. The patient was instructed to apply topical dexamethasone tobramycin for 2 weeks and pranoprofen for 1 month postoperatively.

**Fig. 3:**
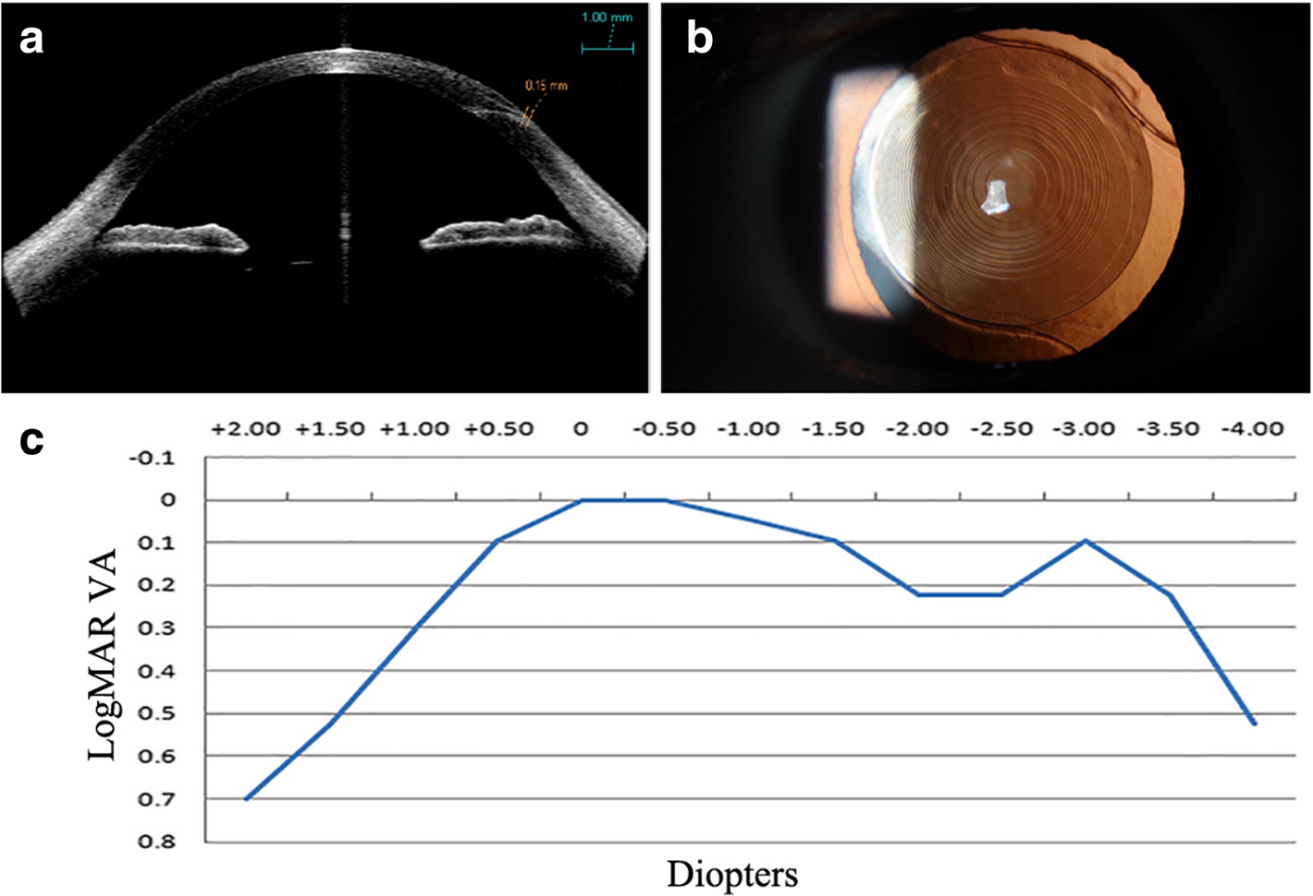
**a** AS-OCT taken at 1 week postoperatively. The distance from the external wound opening to the corneal flap edge was 0.15 mm. Bar,1 mm. **b** Slitlamp examination showed well centered IOL with a 360° overlapping capsular edge at 3 months postoperatively. **c** Defocus curve at 3 months postoperatively

**Fig. 4:**
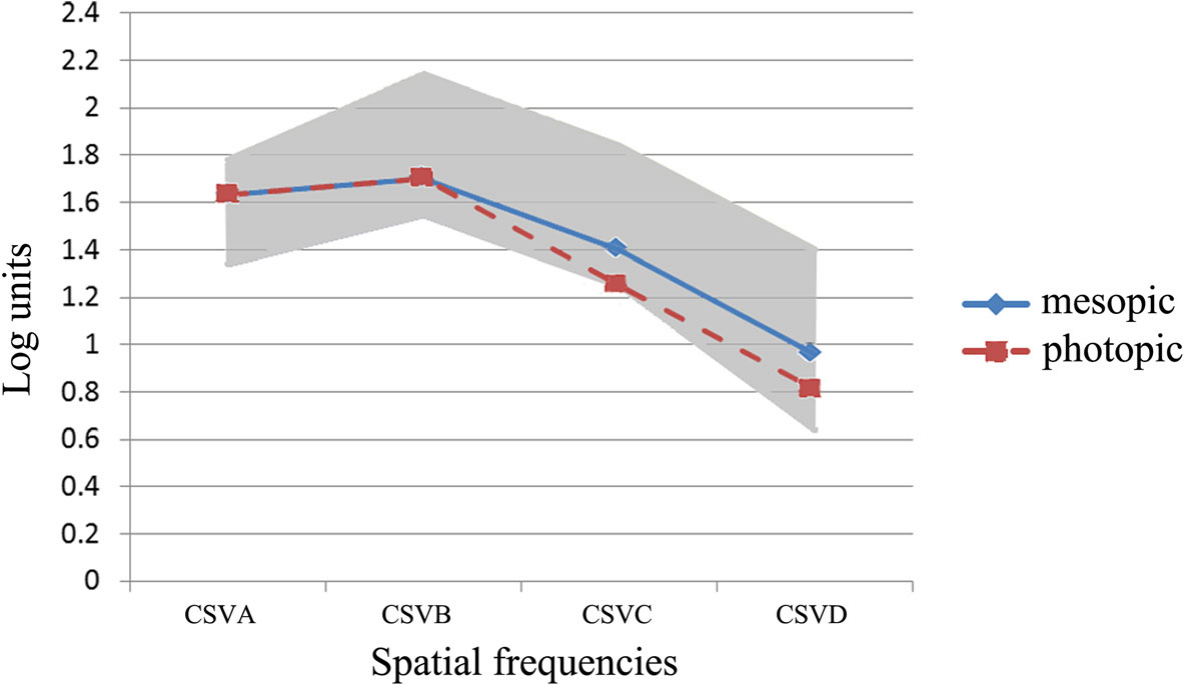
Contrast sensitivity under mesopic and photopic conditions at 3 months postoperatively. Grey area shows the normal range of contrast sensitivity among 56–75 year-old people

Anterior segment optical coherence tomography (AS-OCT, Carl Zeiss Meditec) showed a smooth corneal flap 1 week after FLACS (Fig. 3a). The distance from the external wound opening to the corneal flap edge was 0.15 mm. At 3 months postoperatively, the IOL was well centered in the capsule (Fig. 3b). Pentacam showed a uniform corneal flap (Fig. 1c, d) with slightly decreased total corneal astigmatism and corneal SA (Table 1). Uncorrected visual acuitis were 0.00 LogMAR for distance, 0.10 LogMAR for intermediate at 80 cm, 0.10 LogMAR for near at 40 cm. The defocus curve (Fig. 3c) showed an optimal visual acuity at -3D apart from 0D, but maintained a functional range of visual acuity across from 0D to − 3.5D with visual acuity no less than 0.22 logMAR. Results of ocular aberrations (OPD Scan, Nidek Co., Ltd.) for 5 mm diameter pupils showed 0.831um of high order aberration (HOA), 0.648um of coma, 0.327um of trefoil, 0.119um of tetrafoil, and 0.311um of SA. Contrast sensitivity (CS, CSV-1000, Vector Vision, Greenville, OH) at 4 spatial frequencies (A: 3 cpd, B: 6 cpd, C:12 cpd and D:18 cpd) under both mesopic (3 cd/m^2^) and photopic (85 cd/m^2^) conditions were at a relatively low level within the normal range (Fig. 4). Despite a mild halo, the patient was very satisfied with his vision.

**Table 1:** Preoperative and postoperative pentacam measurements

	Preoperative	Postoperative	
		1 week	3 months
K1	37.5@49.6	37.2@56.2	37.7@69.2
K2	38.4@139.6	37.6@146.2	38.2@159.2
Total Astig(D)	0.9	0.4	0.5
SA at 6 mm zone(um)	0.392	0.305	0.375
Irregular astig(um)	0.115	0.238	0.161

Pentacam showed slightly decreased postoperative total corneal astigmatism and corneal SA. Besides, there was only a slight change in astigmatism and axial direction at 3 months compared to that of 1 week postoperative

## Discussion

The application of femtosecond laser (FSL) in cataract surgery improves the precision of corneal incision location and extent. FSL also provides a precise and well-centered capsulotomy which contributes to the proper position of the IOL that may be related to refractive outcome improvements [4]. In this patient, the computer-controlled accurate steep merdian incision successfully decreased the total corneal astigmatism and ensured visual outcomes of this patient. Well-centered trifocal IOL with a 360° overlapping capsular edge could reduce the incidence of myopization and HOAs changes. In post-LASIK eyes, postoperative corneal edema after cataract surgery may accumulate in the flap interface and the flap itself, causing early transient central corneal steepening and consequent myopic shift that could disappear after the corneal edema resolves [1]. However, the incidence of this complication could be reduced by FSL assisted lens fragmentation, which helps reduce phacoemulsification energy requirements, protect corneal endothelial cells, and thus shorten the recovery period and improve visual outcomes [5].

As for safety, although vacuum suction during FLACS can lead to an increase of the intraocular pressure, it was proved to be feasible for those with previous corneal surgeries, such as radial keratotomy [6] or even penetrating keratoplasty [7]. In this patient, the docking and suction procession did not have any negative effect on the corneal flap, and no intraoperative complications were observed throughout the surgery either. However, the AS-OCT showed a very short distance between the external wound opening and the corneal flap edge postoperatively. Zhu et al. found that laser corneal incisions were closer to the center of the cornea than manual corneal incisions, and they thought this may be related to the possible inaccuracy or uncertainty in corneal incision positioning of the LenSx machine [8]. So here we have to point out that a laser-assisted corneal incision may increase the risk of intersection between the incision and the corneal flap. Future studies should be conducted to confirm this hypothesis.

Post-LASIK patients are often focusing on better visual quality and they are keen to take off spectacles, even after developing cataract. A few studies have reported that the implantation of multifocal IOLs in patients who underwent previous myopic LASIK provided good visual acuities for distance and near range [9]. The AT LISA tri839MP, a diffractive trifocal preloaded IOL with an asphericity of − 0.18, provides a near addition (add) of + 3.33 D and an intermediate add of + 1.66D. It was developed to overcome the photic phenomena and the poor level of intermediate vision of traditional multifocal IOLs [10]. It is well known that laser refractive surgery would modify the corneal shape and induce positive spherical aberration (SA) while correcting myopia [11]. The negative SA of AT LISA tri839MP could partially compensate SA and help retain good mesopic distance vision.

Furthermore, thorough preoperative evaluation was fundamental for the success of this operation. The inclusion criteria for multifocal IOL implantation in our eye center are corneal astigmatism of no more than 1.25D, root mean square (RMS) of corneal HOAs within 6.0 mm zone no more than 0.50 mm, and kappa angle no more than 0.29. The criteria are consistent with those in the studies of Monaco [12] and Mojzis [9]. In this case, the patient met all the above requirements despite of his myopic LASIK history. In this case, we propose that post-LASIK eye necessitates a uniform, well-centered and closely-attached corneal flap for multifocal IOL implantation. Another noticeable situation is the possible aggravation of dry eye after FLACS [13], since that LASIK procedure had already potentiate the instability of tear film. Therefore, careful evaluation of dry eye and timely treatment before surgery is recommended.

IOL power calculation remains one of the most difficult parts for cataract operation after refractive surgery. In Wong’s study, the Asian eyes with previous myopic LASIK or photorefractive keratectomy (PRK) had cataract surgeries with IOL power calculated by 4 formulas. Haigis-L formula turned out to have the highest percentage of cases that achieved the refraction of targeted SE ±0.50 D and SE ±1.00 D [14]. A meta-analysis by Chen et al. also concluded that the clinical inquiry was inaccurate in predicting postoperative refraction as compared to the Haigis-L formula [15]. In this case, the Haigis-L formula again proved to be reliable to attain emmetropia.

## Conclusion

In conclusion, our report shows that, for cataract patients with previous LASIK, FLACS with implantation of the AT LISA tri839MP can be an effective option to obtain spectacle independence.

## Abbreviations

AS-OCT: Anterior segment optical coherence tomography
CDVA: Corrected distance visual acuity
CS: Contrast sensitivity
FLACS: Femtosecond laser-assisted cataract surgery
FSL: Femtosecond laser
HOAs: Higher-order aberrations
IOL: Intraocular lens
LASIK: Laser in situ keratomileusis
PRK: Photorefractive keratectomy
SA: Spherical aberration
VA: Visual acuity

## References

[1] Alio JL, Abdelghany AA, Abdou AA, Maldonado MJ (2016). Cataract surgery on the previous corneal refractive surgery patient. Surv Ophthalmol.

[2] Grewal DS, Schultz T, Basti S, Dick HB (2016). Femtosecond laser-assisted cataract surgery--current status and future directions. Surv Ophthalmol.

[3] de Vries NE, Nuijts RM (2013). Multifocal intraocular lenses in cataract surgery: literature review of benefits and side effects. J Cataract Refract Surg.

[4] Nagy ZZ, McAlinden C (2015). Femtosecond laser cataract surgery. Eye Vis.

[5] Chen X, Yu Y, Song X, Zhu Y, Wang W, Yao K (2017). Clinical outcomes of femtosecond laser-assisted cataract surgery versus conventional phacoemulsification surgery for hard nuclear cataracts. J Cataract Refract Surg.

[6] Noristani R, Schultz T, Dick HB (2016). Femtosecond laser-assisted cataract surgery after radial keratotomy. J Refract Surg.

[7] Cao D, Wang S, Wang Y. Femtosecond laser-assisted cataract surgery after penetrating keratoplasty: a case report. BMC Ophthalmol. 2017;17(1) 10.1186/s12886-017-0496-1 PMC5483306 28646864

[8] Zhu S, Qu N, Wang W, Zhu Y, Shentu X, Chen P, Xu W, Yao K (2017). Morphologic features and surgically induced astigmatism of femtosecond laser versus manual clear corneal incisions. J Cataract Refract Surg.

[9] Chang JS, Ng JC, Chan VK, Law AK (2017). Visual outcomes, quality of vision, and quality of life of diffractive multifocal intraocular Lens implantation after myopic laser in situ Keratomileusis: a prospective, observational case series. J Ophthalmol.

[10] Mojzis P, Majerova K, Hrckova L, Pinero DP (2015). Implantation of a diffractive trifocal intraocular lens: one-year follow-up. J Cataract Refract Surg.

[11] Iijima K, Kamiya K, Shimizu K, Igarashi A, Komatsu M (2015). Demographics of patients having cataract surgery after laser in situ keratomileusis. J Cataract Refract Surg.

[12] Monaco G, Gari M, Di Censo F, Poscia A, Ruggi G, Scialdone A (2017). Visual performance after bilateral implantation of 2 new presbyopia-correcting intraocular lenses: trifocal versus extended range of vision. J Cataract Refract Surg.

[13] Yu YH, Hua HX, Wu MH, Yu YB, Yu WS, Lai KR, Yao K (2015). Evaluation of dry eye after femtosecond laser-assisted cataract surgery. J Cataract Refract Surg.

[14] Wong CW, Yuen L, Tseng P, Han DC (2015). Outcomes of the Haigis-L formula for calculating intraocular lens power in Asian eyes after refractive surgery. J Cataract Refract Surg.

[15] Chen X, Yuan F, Wu L (2016). Metaanalysis of intraocular lens power calculation after laser refractive surgery in myopic eyes. J Cataract Refract Surg.

